# Influence of various pilot hole profiles on pedicle screw fixation strength in minimally invasive and traditional spinal surgery: a comparative biomechanical study

**DOI:** 10.3389/fbioe.2024.1359883

**Published:** 2024-02-06

**Authors:** Yun-Da Li, Po-Liang Lai, Ming-Kai Hsieh, Weng-Pin Chen, De-Mei Lee, Tsung-Ting Tsai, Ching-Lung Tai

**Affiliations:** ^1^ Department of Biomedical Engineering, Chang Gung University, Taoyuan, Taiwan; ^2^ Department of Orthopaedic Surgery, Spine Section, Bone and Joint Research Center, Chang Gung Memorial Hospital and Chang Gung University College of Medicine, Taoyuan, Taiwan; ^3^ Department of Orthopedic Surgery, New Taipei Municipal TuCheng Hospital (Built and Operated by Chang Gung Medical Foundation), New Taipei City, Taiwan; ^4^ Department of Mechanical Engineering, National Taipei University of Technology, Taipei, Taiwan; ^5^ Department of Mechanical Engineering, Chang Gung University, Taoyuan, Taiwan

**Keywords:** pedicle screw, screw shape, pilot hole profile, screw pullout test, minimally invasive spinal surgery, traditional spinal surgery

## Abstract

Despite advancements in pedicle screw design and surgical techniques, the standard steps for inserting pedicle screws still need to follow a set of fixed procedures. The first step, known as establishing a pilot hole, also referred to as a pre-drilled hole, is crucial for ensuring screw insertion accuracy. In different surgical approaches, such as minimally invasive or traditional surgery, the method of creating pilot holes varies, resulting in different pilot hole profiles, including variations in size and shape. The aim of this study is to evaluate the biomechanical properties of different pilot hole profiles corresponding to various surgical approaches. Commercially available synthetic L4 vertebrae with a density of 0.16 g/cc were utilized as substitutes for human bone. Four different pilot hole profiles were created using a 3.0 mm cylindrical bone biopsy needle, 3.6 mm cylindrical drill, 3.2–5.0 mm conical drill, and 3.2–5.0 mm conical curette for simulating various minimally invasive and traditional spinal surgeries. Two frequently employed screw shapes, namely, cylindrical and conical, were selected. Following specimen preparation, screw pullout tests were performed using a material test machine, and statistical analysis was applied to compare the mean maximal pullout strength of each configuration. Conical and cylindrical screws in these four pilot hole configurations showed similar trends, with the mean maximal pullout strength ranking from high to low as follows: 3.0 mm cylindrical biopsy needle, 3.6 mm cylindrical drill bit, 3.2–5.0 mm conical curette, and 3.2–5.0 mm conical drill bit. Conical screws generally exhibited a greater mean maximal pullout strength than cylindrical screws in three of the four different pilot hole configurations. In the groups with conical pilot holes, created with a 3.2–5.0 mm drill bit and 3.2–5.0 mm curette, both conical screws exhibited a greater mean maximal pullout strength than did cylindrical screws. The strength of this study lies in its comprehensive comparison of the impact of various pilot hole profiles commonly used in clinical procedures on screw fixation stability, a topic rarely reported in the literature. Our results demonstrated that pilot holes created for minimally invasive surgery using image-guided techniques exhibit superior pullout strength compared to those utilized in traditional surgery. Therefore, we recommend prioritizing minimally invasive surgery when screw implantation is anticipated to be difficult or there is a specific need for stronger screw fixation. When opting for traditional surgery, image-guided methods may help establish smaller pilot holes and increase screw fixation strength.

## 1 Introduction

Frequently employed in the treatment of various spinal pathologies, posterior instrumentation with a pedicle screw-rod construct serves the vital purposes of stabilizing vertebrae, rectifying axial alignment, and achieving spinal fusion (Castro et al., 1976; Boos and Webb, 1997; Gaines, 2000; Ringel et al., 2006; Gautschi et al., 2011; Cheng et al., 2013). Regarding the appearance, thread design, and material of pedicle screws, many innovative and diverse studies have been performed recently (Shea et al., 2014; Hsieh et al., 2020; Liu et al., 2020; Viezens et al., 2021; Tai et al., 2022). However, the standard steps for implanting pedicle screws during surgery still need to follow a fixed protocol (Perna et al., 2016). The first step is to establish a pilot hole, also known as a pre-drilled hole, and the direction and depth of this pilot hole should match those of the implanted screw. This ensures that the screw is subsequently inserted forward in the same direction without any deviation to avoid damaging bony structures such as the pedicle and vertebral body, as well as neural structures within the spinal canal, and to achieve sufficient fixation stability.

The size of the pilot hole affects the process of pedicle screw implantation. If the pilot hole is excessively large, exceeding the inner diameter of the screw, there will be a reduced contact area between the screw thread and the bone, a decrease in bone volume within the screw threads, and, consequently, a reduction in both the insertion torque and pullout strength (Silva et al., 2013). In contrast, a pilot hole that is too small will lead to excessive insertion torque during screw insertion, potentially posing a risk of pedicle fracture, especially in patients with osteoporosis (Shea et al., 2014). In a biomechanical study (Battula et al., 2008), different sizes of pilot holes were compared in an attempt to determine the proper pilot hole size, namely, the critical pilot hole size. With this pilot hole size, a balance between lower insertion torque and higher pullout strength can be achieved. The study showed that a pilot hole size of 71.5% of the screw’s outer diameter meets the expectations of this critical pilot hole size.

**FIGURE 1:**
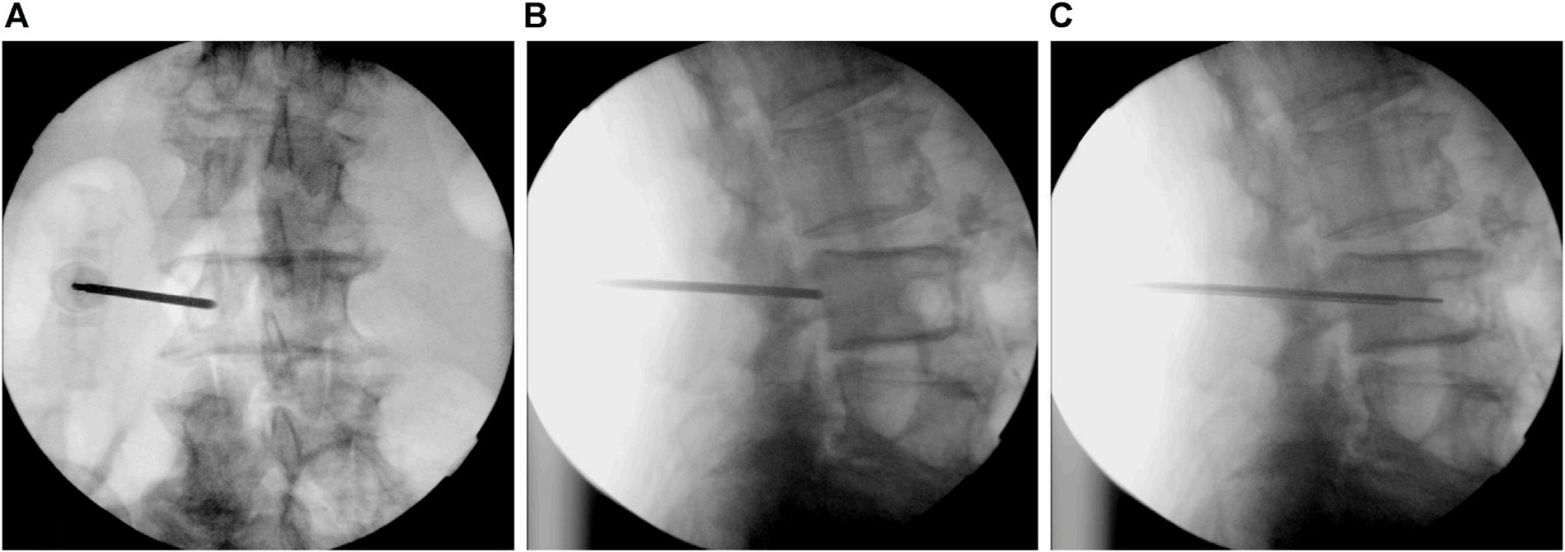
Intraoperative fluoroscopic images used to identify pedicle positions in real-time during the surgery. **(A)** The anteroposterior view shows the biopsy needle entering from the outer edge to the inner edge of the pedicle. **(B)** The lateral view confirms the position of the front end of the biopsy needle within the pedicle. **(C)** The biopsy needle gradually advances into the vertebral body, inserting a guide wire along its hollow interior. Subsequently, a pedicle screw will be implanted using this guide wire.

Posterior lumbar spinal surgeries can be categorized into traditional and minimally invasive surgeries, depending on the different surgical approaches. Traditional surgeries use a midline incision, while minimally invasive surgeries utilizes a paraspinal approach, also known as the Wiltse approach (Wiltse and Spencer, 1988; Vialle et al., 2005; Vialle et al., 2006), which reduces muscle damage in the surgical field. These two surgical approaches use different methods for inserting pedicle screws. One is the traditional freehand technique (Karapinar et al., 2008; Mattei et al., 2009; Vijayeswaran et al., 2019; He et al., 2022), and the other is the minimally invasive image-guided technique (Gelalis et al., 2012; Lieberman et al., 2020; Sun et al., 2020; Li et al., 2022; Zhang et al., 2023). These two techniques also correspond to different pilot hole profiles. When using the traditional freehand technique to insert screws, a pedicle probe or curette is employed to create a conical pilot hole. In contrast, minimally invasive surgery, guided by imaging techniques such as fluoroscopy, navigation, or robotic arms, involves the use of a Jamshidi biopsy needle or drill bit to create a pilot hole. The image-guided techniques utilize intraoperative fluoroscopy or computed tomography to identify pedicle positions in real-time during the procedure, guiding the insertion of Jamshidi biopsy needles or drill bits (Figure 1). The biopsy needle does not remove bone during the process, while the drill generates bone shavings during the procedure, but both result in a cylindrical pilot hole. Furthermore, during the creation of a conical pilot hole using a curette, it is hypothesized that there may be a compressive effect on the surrounding bone. Whether this effect can enhance the stability of pedicle screw fixation is also a topic of interest.

Past studies on pilot holes have focused primarily on their size (Oktenoglu et al., 2001; Battula et al., 2008; Chatzistergos et al., 2010; Kunkel et al., 2011; Silva et al., 2013; Shea et al., 2014; Prasad et al., 2016), with little exploration of other pilot hole characteristics, such as shape and the impact of bone removal. Therefore, we conducted this experiment using synthetic bone material and different shapes of pedicle screws to investigate the biomechanical performance of various pilot hole configurations.

## 2 Materials and methods

### 2.1 Synthetic bone specimens and pedicle screw geometries

As the focus of this study is closely related to morphology, bone specimens closely resembling the actual human anatomical structure were required. Thus, this study utilized commercially available synthetic L4 vertebrae (Model #3429-4-2, Pacific Research Laboratory, Inc., Vashon Island, WA, United States) to simulate normal spinal vertebrae, and simultaneously reduce variations in shape, dimensions, bone density, and other factors among specimens. These synthetic vertebrae were constructed with solid foam cancellous cores at a density of 0.16 g/cc, ensuring a consistent and uniform morphometry comparable to that of human vertebrae.

**FIGURE 2:**
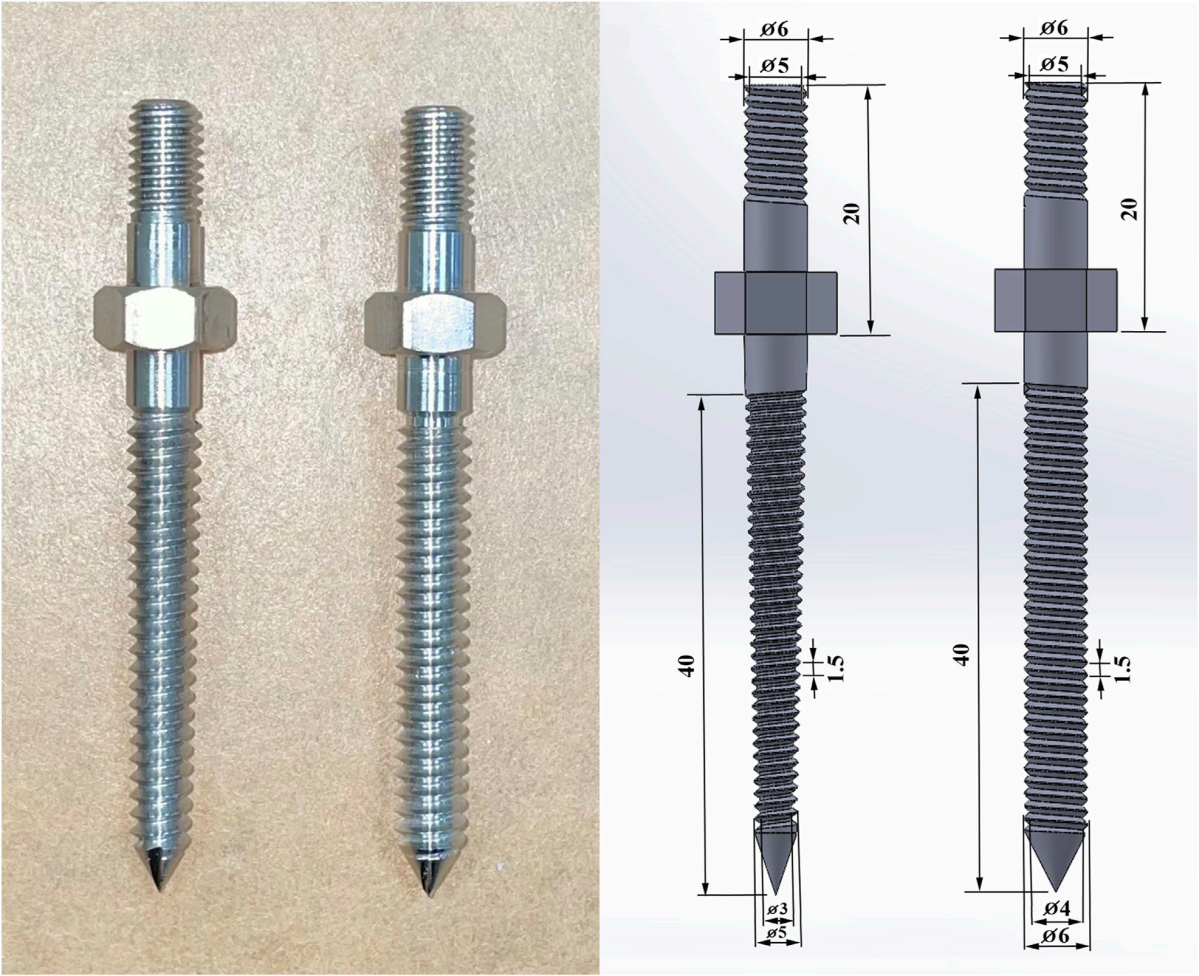
Photographs and schematic depictions of the pedicle screws, including the conical and cylindrical shapes. The conical screw was tapered, reducing in diameter from 6 mm at the hub to 5 mm at the tip, whereas the cylindrical screw maintained a steady diameter of 6 mm along its length.

As the experiment explored the impact of various pilot hole shapes, distinct types of screws were chosen to complement the study, examining the interactions between pilot holes of different shapes and their corresponding screws. The screws were classified as cylindrical, with a consistent diameter of 6 mm, or conical, featuring a diameter tapering from 6 mm at the hub to 5 mm at the tip. Both screw designs featured a thread depth of 1 mm and a thread pitch of 1.5 mm. Additionally, the standardized thread coverage length was set at 40 mm. Figure 2 provides schematic depictions of the pedicle screws.

### 2.2 Preparation of various pilot hole profiles

**FIGURE 3:**
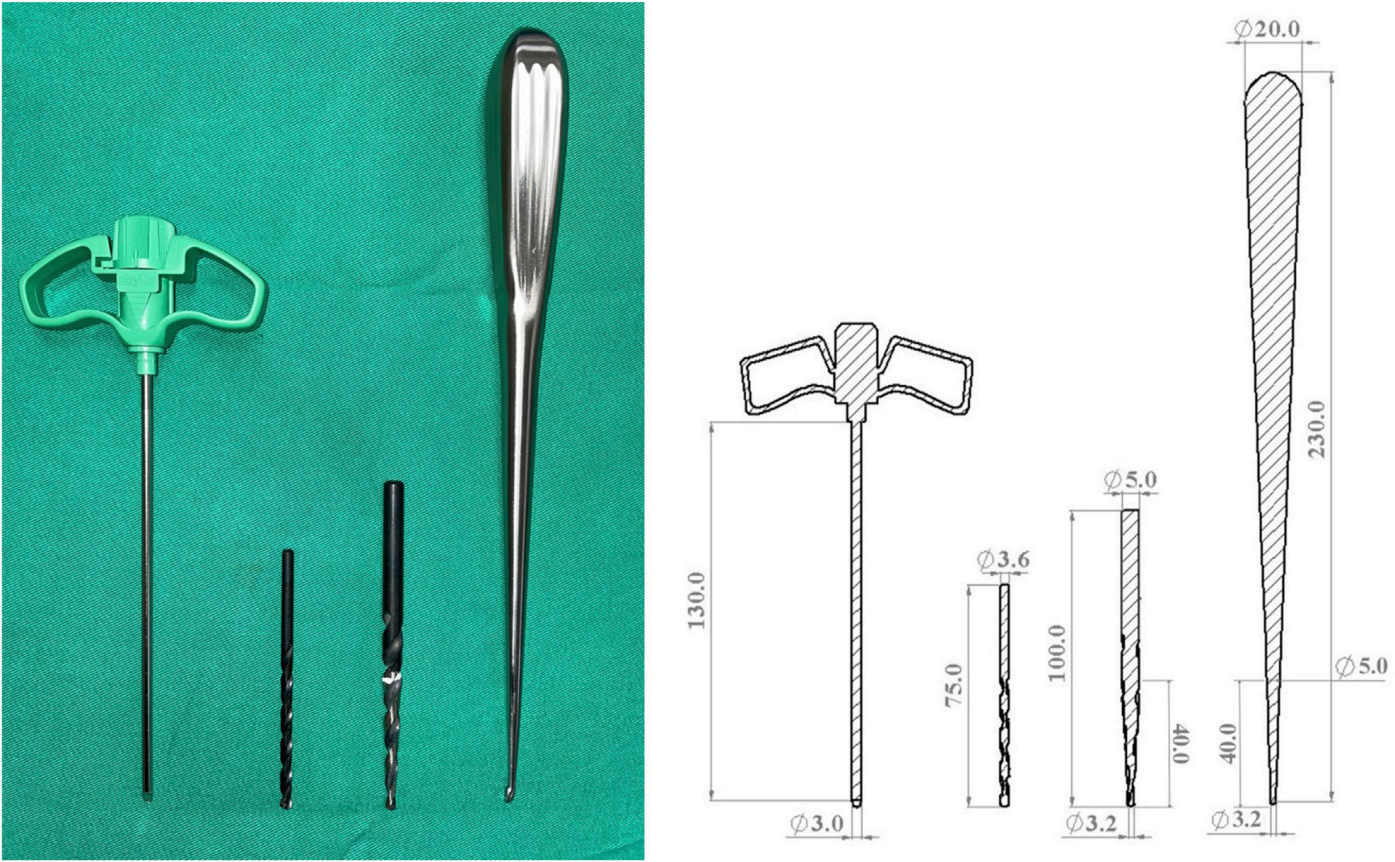
Photographs and schematic representations of the four different pilot hole profiles, including those of a 3 mm cylindrical biopsy needle, a 3.6 mm cylindrical drill bit, a 3.2–5 mm conical drill bit, and a 3.2–5 mm conical curette.

To emulate the diverse pilot hole configurations utilized in surgical procedures, biopsy needles, curettes, and drill bits with distinct diameters and shapes were employed. The first type of pilot hole was created using a cylindrical Jamshidi biopsy needle (Stryker Corp., Airview Boulevard Kalamazoo, MI, United States) with a diameter of 3 mm (3 mm biopsy needle), simulating the process of pilot hole creation in minimally invasive surgery guided by fluoroscopy. The second type of pilot hole was made with a cylindrical drill bit with a diameter of 3.6 mm (3.6 mm drill bit), emulating the process of creating pilot holes in minimally invasive surgery using navigation systems or robotic arms. The third type of pilot hole involved the use of a conical drill bit with a tip diameter of 3.2 mm and a proximal diameter of 5.0 mm (3.2–5 mm drill bit). In the fourth type, a cylindrical drill bit with a diameter of 3.2 mm was initially utilized to create a pathway. Subsequently, a curette was employed to gradually deepen the hole along this pathway, creating a conical pilot hole. These steps represent the practical approach applied in traditional spine surgery. The curette had a tip diameter of 3.2 mm, and at a distance of 40 mm from the distal end, the diameter increased to 5 mm, matching the dimensions of the third conical drill bit used (3.2–5 mm curette). The depth of each of these four different pilot holes was 40 mm, equivalent to the total length of the screw threads. Figure 3 shows schematic representations of the four different pilot hole profiles.

### 2.3 Allocation of the specimens

The allocation of specimens to experimental groups is presented in Table 1. A total of eight combinations of screw design and pilot hole profile were tested (six replicates in each group).

**TABLE 1:** Allocation of the specimens to experimental groups.

Group	Pilot hole profile	Screw shape	Specimen number
1	3 mm cylindrical biopsy needle	Cylindrical	6
2	3.6 mm cylindrical drill bit	Cylindrical	6
3	3.2–5 mm conical drill bit	Cylindrical	6
4	3.2–5 mm conical curette	Cylindrical	6
5	3 mm cylindrical biopsy needle	Conical	6
6	3.6 mm cylindrical drill bit	Conical	6
7	3.2–5 mm conical curette	Conical	6
8	3 mm cylindrical biopsy needle	Conical	6

### 2.4 Experimental procedures

**FIGURE 4:**
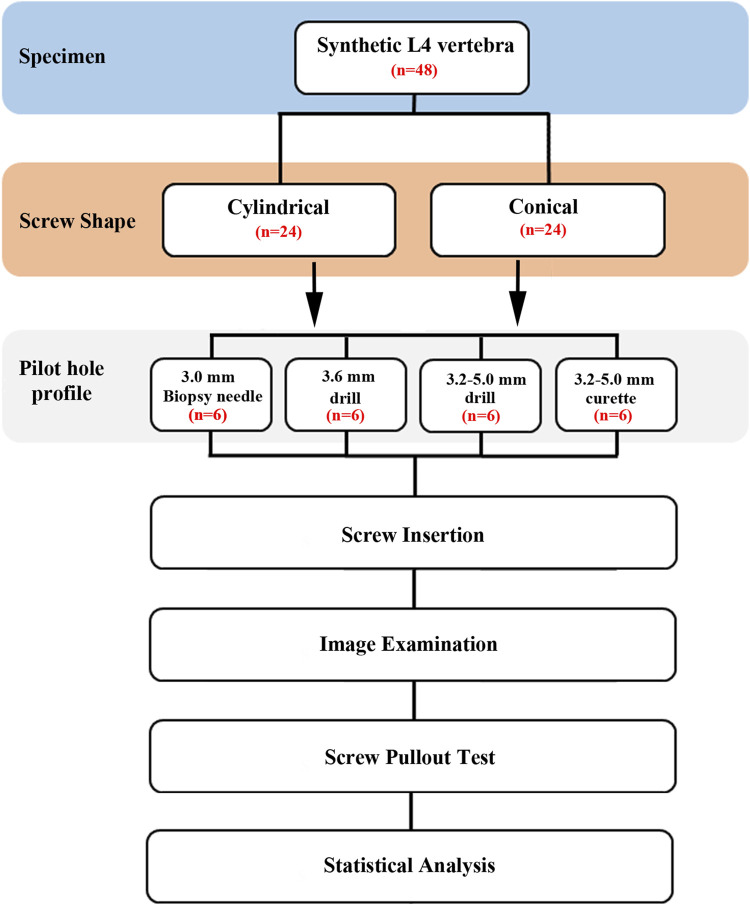
Flowchart of the experimental design. This experiment involved two different shapes of screws and four types of pilot hole profiles.

This study employed synthetic L4 vertebrae as substitutes for typical human vertebral bone. Initially, pilot holes were created in the dorsal cortex of the pedicle using the four methods described above. Subsequently, screws were inserted along these pilot holes until all the threads were fully embedded in the specimen. Due to the irregular shape of the synthetic L4 vertebrae, after screw insertion, the instrumented vertebral specimens were embedded in acrylate resin (#20-3568; Buehler, Lake Bluff, IL, United States) for clamping purposes. The posterior elements of the vertebrae were left unembedded to facilitate subsequent mechanical testing. This experiment involved two different shapes of screws and four types of pilot hole profiles. The experimental flowchart is shown in Figure 4. Each experimental configuration was tested six times for subsequent statistical analysis.

### 2.5 Imaging analysis and biomechanical pullout testing

**FIGURE 5:**
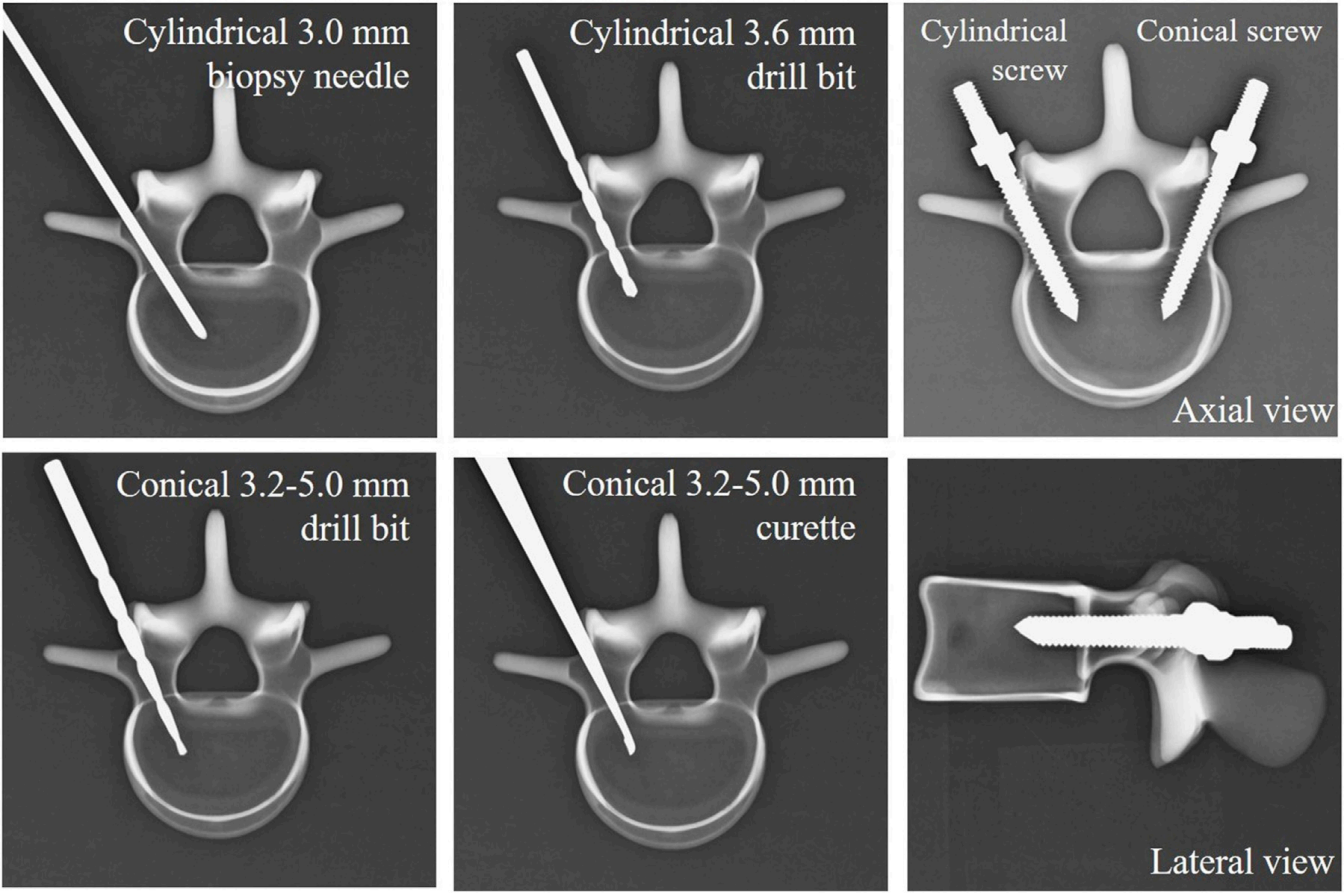
Radiological images of the four types of pilot holes and two types of screws to confirm a suitable trajectory and insertion depth for the pilot hole creation methods and bone screws.

Before conducting the pullout test, we examined axial and sagittal X-ray images (GE DX300 X-ray machine, Salt Lake City, UT, United States) of all specimens. This examination aimed to confirm the suitable trajectory and insertion depth for the four pilot hole creation methods and screws, as depicted in Figure 5. Additionally, a thorough examination of the specimens was carried out to eliminate the possibility of any fractures or defects resulting from screw insertion.

**FIGURE 6:**
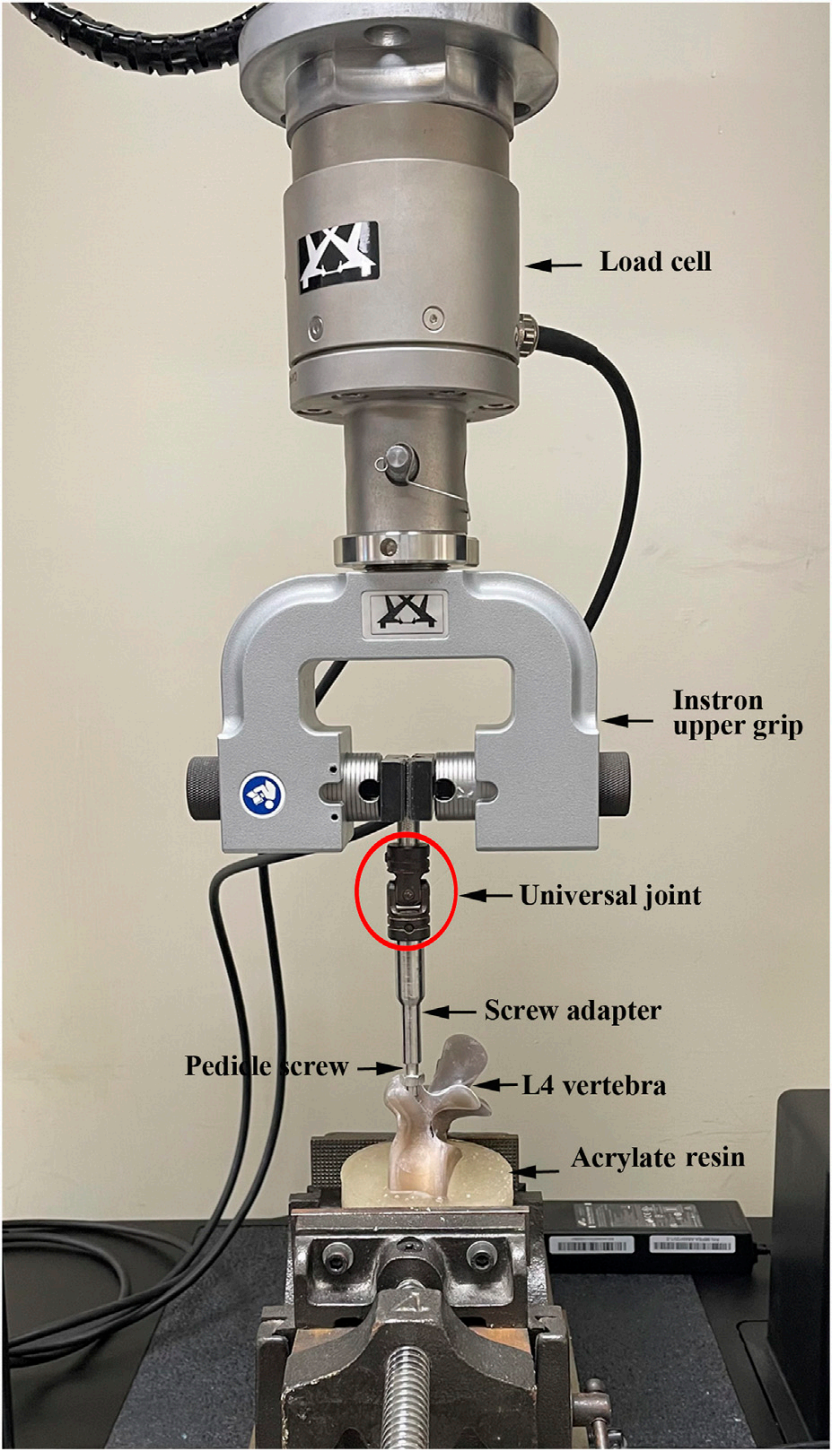
Photographs showing the experimental setup of the screw pullout test. The pedicle screw was attached to a 10-mm cylindrical adapter with a universal joint that automatically adjusted to align the long axis of the screw with the pullout ram of the testing machine, and this adapter was secured to the upper wedge grip of the testing machine.

The procedure for the biomechanical screw pullout test closely resembled the approach utilized in our prior studies (Liu et al., 2020; Hsieh et al., 2022). After screws insertion and specimen embedding in acrylate resin, each prepared specimen was affixed to a custom-made grip positioned on the testing machine platform (68TM-30, Instron Co., Norwood MA, United States). The pedicle screw was attached to a 10-mm cylindrical adapter with a universal joint that automatically adjusted to align the long axis of the screw with the pullout ram of the testing machine, and this adapter was secured to the upper wedge grip of the testing machine. Following the arrangement of the specimen for the experiment, a pullout force was applied to the screw head at a constant rate of 5 mm/min. Throughout the pullout test, the relationship between the applied force and displacement was continuously recorded in 0.05-mm increments until failure. The maximum pullout strength was defined as the peak value recorded during the testing period. The experimental setup for the screw pullout test on standard L4 vertebral specimens is illustrated in Figure 6.

### 2.6 Statistical analysis

To evaluate the influence of the various pilot hole profiles, we statistically compared the ultimate pullout strength of the pedicle screws across two different shapes of screws and four types of pilot holes. The measurements are presented as the mean ± standard deviation (SD), and statistical analyses were conducted using SPSS software (SPSS for Windows version 20, SPSS, Inc., Chicago, IL, United States). Group differences were evaluated through Mann‒Whitney U tests, with a significance threshold set at *p* < 0.05.

## 3 Results

### 3.1 Mean maximal pullout strength of four different types of pilot holes

In the first type of pilot hole, simulating the process of pilot hole creation in minimally invasive surgery guided by fluoroscopy, a cylindrical biopsy needle with a diameter of 3 mm was utilized. The mean maximal pullout strength was 1302.33 ± 64.50 N for conical screws and 1496.24 ± 181.80 N for cylindrical screws.

In the second type of pilot hole, to emulate the process of creating pilot holes in minimally invasive surgery using navigation systems or robotic arms, a cylindrical drill bit with a diameter of 3.6 mm was used. The mean maximal pullout strength was 1236.80 ± 179.51 N for conical screws and 1055.55 ± 62.59 N for cylindrical screws.

In the third and fourth types of pilot holes, a conical drill bit with a tip diameter of 3.2 mm and a proximal diameter of 5 mm, along with a curette of the same diameter within the experimental range, was used to represent the practical approach applied in traditional spinal surgery. The mean maximal pullout strength for conical screws in the third and fourth pilot hole types was 793.21 ± 107.49 N and 1018.37 ± 184.48 N, respectively, while the corresponding values for cylindrical screws were 726.65 ± 87.76 N and 905.99 ± 175.60 N, respectively.

### 3.2 Effect of the pilot hole configuration

**FIGURE 7:**
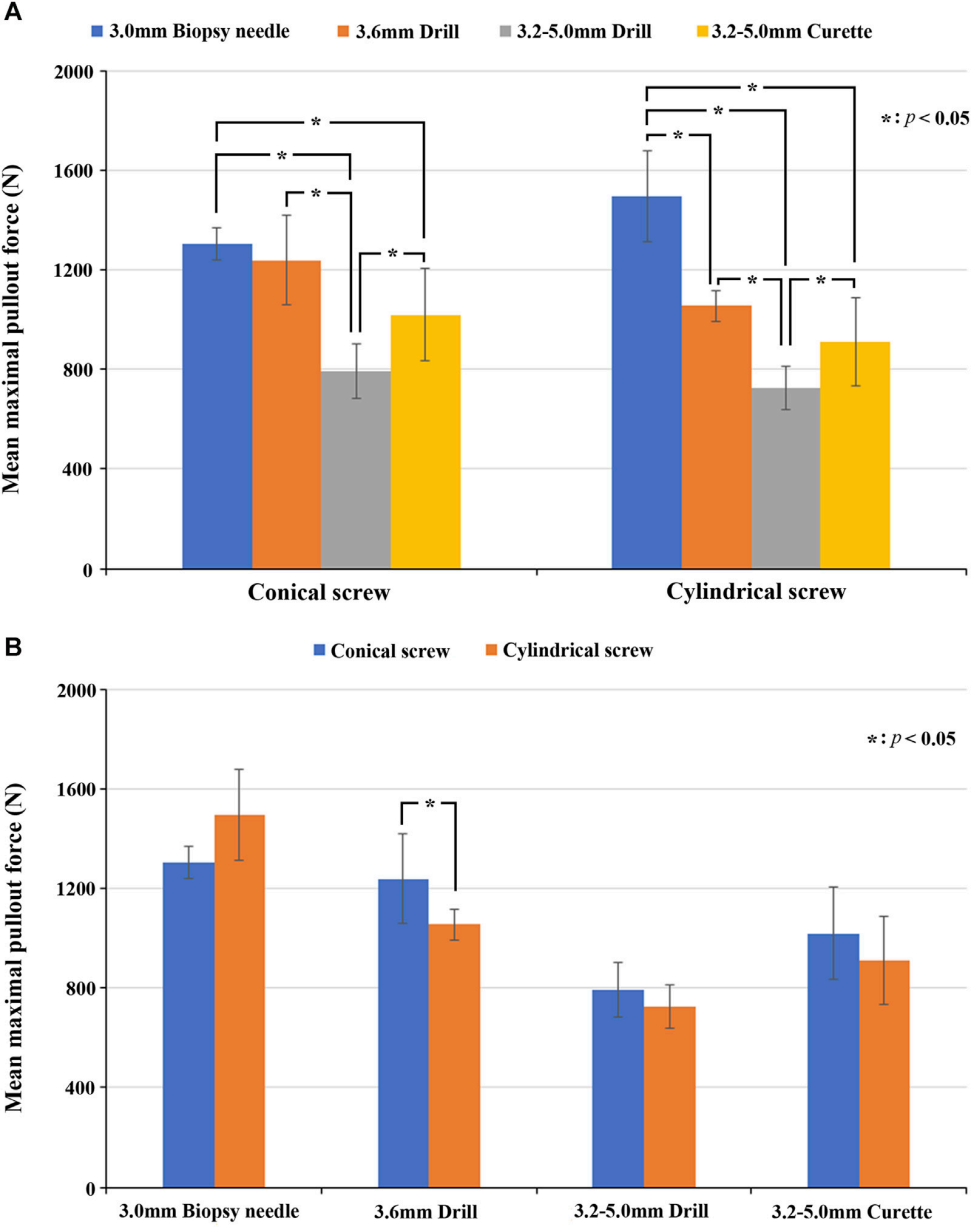
**(A)** Comparisons of the mean maximal pullout strength for four different pilot holes and conical and cylindrical screws. Significant differences between groups are indicated with the “*” symbol. **(B)** Comparisons of the mean maximal pullout strength of the two different screw shapes in each of the four pilot hole profiles. Significant differences between groups are indicated with the “*” symbol.

In both the conical and cylindrical screw groups, the mean maximal pullout strength of the four pilot holes showed a similar trend. The descending order of the mean maximal pullout strength was as follows: 3.0 mm biopsy needle, 3.6 mm drill bit, 3.2–5.0 mm curette, and 3.2–5.0 mm drill bit. Figure 7A shows comparisons of the mean maximal pullout strength for the conical and cylindrical screws in the four different pilot holes.

In the conical screw group, the mean maximal pullout strength with the 3.0 mm drill bit was greater than that with the 3.6 mm drill bit, but without a significant difference (*p* = 0.310); however, it was significantly greater than that with the 3.2–5.0 mm drill bit (*p* = 0.002) and the 3.2–5.0 mm curette (*p* = 0.002). The mean maximal pullout strength with the 3.6 mm drill bit was significantly greater than that with the 3.2–5.0 mm drill bit (*p* = 0.002), and while it was also greater than that with the 3.2–5.0 mm curette, the difference was not significant (*p* = 0.065). The mean maximal pullout strength with the 3.2–5.0 mm curette was significantly greater than that with the 3.2–5.0 mm drill bit (*p* = 0.026).

In the cylindrical screw group, the mean maximal pullout strength with the 3.0 mm biopsy needle was significantly greater than that with the 3.6 mm drill bit (*p* = 0.002), the 3.2–5.0 mm drill bit (*p* = 0.002), and the 3.2–5.0 mm curette (*p* = 0.002). The mean maximal pullout strength with the 3.6 mm drill bit was significantly greater than that with the 3.2–5.0 mm drill bit (*p* = 0.002), and while it was also greater than that with the 3.2–5.0 mm curette, the difference was not significant (*p* = 0.093). The mean maximal pullout strength with the 3.2–5.0 mm curette was significantly greater than that with the 3.2–5.0 mm drill bit (*p* = 0.041).

### 3.3 Effect of the screw shape

Among the various pilot hole groups, the comparative analysis of the mean maximal pullout strength between conical and cylindrical screws as follows. In the 3.0 mm biopsy needle group, the mean maximal pullout strength was greater for cylindrical screws than the conical screws, but the difference was not significant (*p* = 0.093). In the 3.6 mm drill bit group, the mean maximal pullout strength for conical screw was significantly greater than that for cylindrical screws (*p* = 0.041). In both the 3.2–5.0 mm drill bit (*p* = 0.240) and 3.2–5.0 mm curette (*p* = 0.240) groups, the mean maximal pullout strength of conical screws was greater than that of cylindrical screw, but the difference was not significant. The comparisons of the mean maximal pullout strength of the two different screw shapes in each of the four pilot holes are shown in Figure 7B.

### 3.4 Effect of the combination of the pilot hole shape and screw shape

In addition, by analyzing the combination of the pilot hole shape and screw shape, we examined whether these pairings cause any variation in the mean maximal pullout strength. In the cylindrical pilot holes, created with the 3.0 mm biopsy needle and 3.6 mm drill bit, both the conical and cylindrical screws showed a greater mean maximal pullout strength in one of the groups. Specifically, in the 3.0 mm biopsy needle group, the cylindrical screws were superior to the conical screws, although the difference was not statistically significant (*p* = 0.093). In the 3.6 mm drill bit group, the conical screws were significantly superior to the cylindrical screws (*p* = 0.041). In the conical pilot holes, created with the 3.2–5.0 mm drill bit and 3.2–5.0 mm curette, both conical screws exhibited a greater mean maximal pullout strength than the cylindrical screws, but the difference was not significant (*p* = 0.240).

## 4 Discussion

In the process of inserting pedicle screws during spinal surgery, the creation of pilot holes is the first and a crucial step. The quality of these pilot holes significantly affects the safety and stability of subsequently implanted screws. With advancements in various aspects of spinal surgery in recent years, in addition to traditional open surgical approaches, an increasing number of surgeries are now performed using minimally invasive approaches with smaller incisions. In these two types of surgical approaches, different methods are applied for creating pilot holes, involving instruments such as drill bits, pedicle probes and curettes. Consequently, various pilot hole sizes and shapes are generated. We aimed to investigate how factors such as the amount of bone removal during the hole creation process, the combination of different pilot hole and screw shape profiles, and the use of curettes may impact the strength of pedicle screw fixation. Thus, in this study, a biomechanical experiment was performed to investigate the influence of different pilot hole profiles on the maximal pullout strength of pedicle screws. The strength of this study lies in the comprehensive comparison of several pilot hole configurations commonly used in both traditional and minimally invasive spinal surgery currently practiced in clinical settings. The experimental results can indeed be helpful references for spinal surgeons in their selection of surgical approaches and pilot hole creation techniques.

We recognize that screw loosening in clinical settings can be attributed to various factors, including cyclic loading of screws on multiple planes and the biological response of bone to the screws over an extended duration. Nevertheless, screw pullout tests have been widely utilized to assess the effectiveness of new screw designs aimed at improving stability. While axial failure of a screw is infrequent in clinical conditions, the availability and reliability of the screw pullout test make it the most efficient method for evaluating a screw’s fixation stability. In our study, we exclusively used axial screw pullout to assess screw anchoring power, concentrating on axial loading and excluding consideration of complex multidirectional forces. Despite this limitation, the axial screw pullout test is considered an efficient means of comparing relative screw anchoring power after implantation. In our present study, all experimental procedures were performed in a consistent manner to ensure uniformity and reproducibility. According to the literature, numerous studies (Lill et al., 2000; Abshire et al., 2001; Liu et al., 2020; Li et al., 2022; Hsieh et al., 2022) have substantiated the argument that comparing the fixation strength of various screw designs through pullout tests yields reliable experimental results. Therefore, we chose axial pullout testing to analyze screw fixation stability because this method is intuitive and highly reproducible. The results of our experiment indicated that there were indeed differences in the mean maximal pullout strength corresponding to the various pilot hole profiles used in both traditional and minimally invasive spinal surgery, with pilot holes created for minimally invasive surgery using image-guided techniques demonstrated superior mean maximal pullout strength compared to those used in traditional surgery.

In terms of synthetic bones, numerous biomechanical investigations have utilized synthetic standard vertebrae (featuring the integral geometry of a single vertebra) (Hsieh et al., 2019; Liu et al., 2020; Hsieh et al., 2022) and polyurethane (PU) foam blocks (Chatzistergos et al., 2010; Liu et al., 2020; Li et al., 2022; Hsieh et al., 2022) to replicate healthy vertebrae and osteoporotic cancellous bone, respectively. These studies propose that these synthetic bones serve as effective alternatives for *in vitro* experiments. Hsieh et al. (Hsieh et al., 2019) used synthetic standard L4 vertebrae, imitating healthy vertebrae to assess the screw anchoring capability between intact and fractured pedicles. The findings indicated a significant reduction in screw anchoring power in the absence of a pedicle. In cases where the pedicle is broken, the study suggests a revision with a larger or longer-diameter screw. In our recent study (Li et al., 2023), polyurethane (PU) foam blocks were employed to simulate cadaveric bones with osteoporosis, healthy bones, and high bone quality, respectively, to evaluate the biomechanical properties of screw turnback and demonstrate the reduction in the fixation stability after the screw is turned 360° from its full insertion position. The results suggested that pedicle screw turnback after full insertion should be reduced in spinal surgeries, particularly procedures that use conical screws in osteoporotic bone. In the present study, considering the experiment’s incorporation of the pilot hole shapes and pedicle screws, we further utilized synthetic L4 vertebra made up of both cortical bone and cancellous bone, with a density of 0.16 g/cm^3^, instead of polyurethane (PU) foam blocks. This choice better replicated the anatomical structure and approximate bone density of the human body while concurrently minimizing variations in shape, dimensions, bone density, and other factors among specimens. These features provided distinct advantages in utilizing synthetic bone material for the experiment, ensuring consistent and reproducible conduct of all experimental procedures.

In past studies on pilot holes, the focus has primarily been on the impact of pilot hole diameter. Battula et al. (2008) conducted a biomechanical analysis to compare the effects of pilot holes with different diameters on the insertion torque and pullout strength. The aim was to identify the most suitable pilot hole size, referred to as the critical pilot hole size. The study compared four pilot hole sizes, corresponding to 70%, 71.5%, 73%, and 80% of the screw’s outer diameter. The research indicated that when the pilot hole diameter exceeded 71.5% of the screw’s outer diameter, the pullout strength began to decrease. Therefore, this study recommended referring to the pilot hole 71.5% of the screw’s outer diameter in size as the critical pilot hole size. After we applied the results of this study to our research, considering that the screw outer diameter used in our experiment was 60 mm, the critical pilot hole was determined as 4.29 mm. In the two groups in which a 3.2–5 mm drill bit and 3.2–5 mm curette were applied, there was indeed a range of pilot hole diameters exceeding 4.29 mm, resulting in a decreased maximal pullout strength. The other two sets of pilot holes were made with a 3 mm biopsy needle and 3.6 mm drill bit, representing 50% and 60%, respectively, of the screw’s outer diameter. Our findings showed that the smallest pilot hole size, i.e., 50% of the outer diameter, yielded the maximal pullout strength. Battula et al.'s study focused on traditional cortical screws, which differed from our study, involving the implantation of pedicle screws in artificial L4 vertebrae. Based on the results of our study, one may wonder whether a smaller pilot hole size could result in better pullout strength. However, according to clinical experience, if the pilot hole size is too small, the guiding role of the pilot hole may be hindered during screw insertion. Once the screw is inserted into an incorrect path, it may not only damage the normal bone structure, leading to a significant reduction in fixation strength, but also harm neural structures, resulting in side effects. Additionally, a pilot hole that is too small may significantly increase the insertion torque, increasing the risk of pedicle fracture, as mentioned by Shea et al. (2014), especially in patients with osteoporosis. Therefore, the pilot hole size should fall within an appropriate range and should not be excessively large or small.

A *in vivo* biomechanical investigation reported by Silva et al. (2013) revealed that a large pilot hole exceeding the inner diameter of the screw can lead to a decreased interface contact area between the screw and bone, reduced bone volume within the screw threads, and, subsequently, decreased insertion torque and pullout strength. Other studies, conducted by Eriksson and Albrektsson (1983), Togni et al. (2011), allowed for an analysis from the perspective of histomorphometry and provided insights into bone remodeling near the screw after insertion, which resembled the initial stages of fracture healing. When the screw was implanted into a smaller pilot hole, bone healing at the screw-bone interface tended to be more active. The findings of these studies suggest that this difference may be attributed to the amount of compacted bone, microfractures, and bleeding affecting bone remodeling, explaining the greater bone volume around the screw threads in cases of smaller pilot holes.

From the discussions in the aforementioned studies regarding the size of pilot holes, it is evident that selecting an appropriate pilot hole size is a crucial decision, especially when paired with the dimensions of commonly used pedicle screws in such surgeries. Moreover, emphasis should be placed not only on the outer diameter of the screw but also on the inner diameter, which is equally important. The size of the pilot hole should not exceed the inner diameter of the screw, as this would significantly decrease the fixation strength. Taking the screws used in our experiment as examples, the conical screw had an inner diameter ranging from 3 to 4 mm, while the cylindrical screw has an inner diameter of 4 mm. Therefore, our experimental results showed that the optimal pullout strength was achieved through the use of a 3 mm biopsy needle, which did not entirely exceed the inner diameter of the screw. Considering both practical clinical experience and our experimental results, a pilot hole size of approximately 50% of the screw’s outer diameter, usually already smaller than the inner diameter of the screw, would be a reasonable and recommended pilot hole size.

**FIGURE 8:**
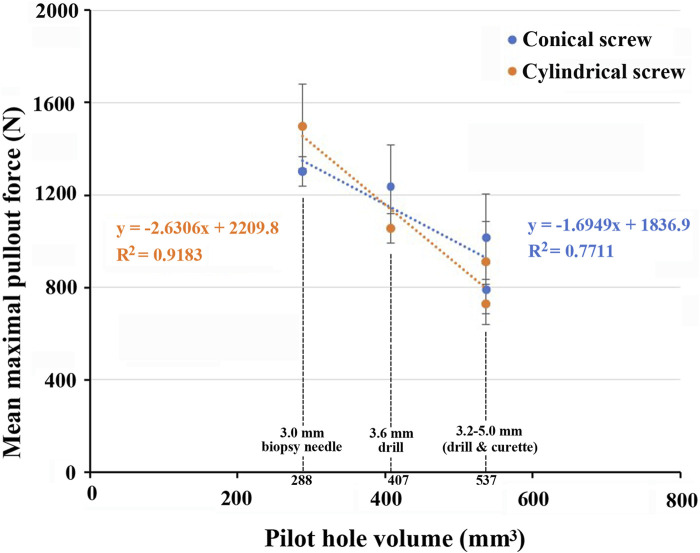
Graph demonstrating an inverse relationship between the pilot hole volume and the mean maximal pullout strength.

In our experiment, the four pilot hole sizes, whether in the conical screw group or the cylindrical screw group, exhibited similar trends in terms of the mean maximal pullout strength. The mean maximal pullout strength in descending order was as follows: 3 mm biopsy needle, 3.6 mm drill bit, 3.2–5 mm curette, and 3.2–5 mm drill bit. We believe that these differences are related to the pilot hole volume and the bone density around the screw. When the pilot hole size is smaller and less bone is removed during the process, after screw insertion, there is more bone around the screw, and the bone volume within the screw threads is denser. Therefore, greater screw pullout strength would be expected. The formula for the volume of the frustum of a cone is $\frac{1}{12}\pi H\left(d^{2}+dD+D^{2}\right)$ , where H is the depth of the pilot hole, d is the diameter at the far end of the pilot hole, and D is the diameter at the near end of the pilot hole. Substituting d = D results in the formula for the volume of a cylinder. The volumes of the four pilot holes were calculated using this formula, resulting in 287.74, 407.15, 536.58, and 536.58 mm³ for the 3 mm biopsy needle, 3.6 mm drill bit, 3.2–5 mm curette, and 3.2–5 mm drill bit, respectively. The distinction between the 3.2–5 mm curette and 3.2–5 mm drill bit lies in the fact that in the 3.2–5 mm curette group, we initially used a 3.2 mm drill bit to create a path and then gradually advanced the curette along this path to exert pressure on the surrounding bone, completing the pilot hole. Consequently, although the volumes of these two pilot holes were identical, it was speculated that the bone density surrounding the 3.2–5 mm curette was greater, resulting in a higher pullout strength in 3.2–5 mm curette group compared to that of 3.2–5 mm drill bit group. These results aligned with our speculation regarding the relationship between the pilot hole volume and pullout strength, demonstrating that as the pilot hole volume increased, the mean maximal pullout strength decreased. Figure 8 illustrates the inverse relationship between the pilot hole volume and the mean maximal pullout strength.

Regarding the shape of the screws, conical screws showed a greater mean pullout strength than did cylindrical screws for three of the four pilot holes. Significant differences were observed, particularly in the 3.6 mm drill bit group, while no significant differences were found in the 3.2–5 mm drill bit or 3.2–5 mm curette groups. In another group utilizing a 3 mm biopsy needle, the mean pullout strength of cylindrical screws was greater than that of conical screws, but the difference was not significant. Overall, conical screws exhibited greater pullout strength than cylindrical screws, consistent with previous findings (Chao et al., 2008; Kim et al., 2012; Liu et al., 2020). Additionally, we combined the shapes of both the pilot hole and screw to analyze the relationship between their configurations and the mean maximal pullout strength. In the groups with cylindrical pilot holes, namely, holes created using a 3 mm biopsy needle or 3.6 mm drill bit, both conical and cylindrical screws showed a greater mean maximal pullout strength in one of the groups. In the groups with conical pilot holes, specifically, holes created using a 3.2–5 mm drill bit or 3.2–5 mm curette, conical screws exhibited a greater mean maximal pullout strength. We observed that, in cylindrical pilot holes, there was not much difference in the mean pullout strength between the two types of screw shapes. However, in conical pilot holes, which simulate pilot holes in traditional spinal surgery, the mean maximal pullout strength of conical screws, with the same shape as the pilot holes, was superior to that of cylindrical screws.

Oikonomidis et al. conducted a biomechanical fatigue test (Oikonomidis et al., 2020), in which two methods of creating pilot holes during surgery were compared: a conical thoracic pedicle probe and a 3.2 mm diameter drill bit. In the experiment, screws were implanted into intact human thoracic spine cadaver bones, followed by fatigue testing of the screws. The results indicated that the choice between using a thoracic pedicle probe or a drill for pilot hole preparation did not seem to affect the rate of screw loosening. The conclusion of the study indicated that using a conical probe for pilot hole preparation might theoretically increase bone density but did not provide biomechanical advantages in terms of the fixation strength of pedicle screws. However, this aspect can be clarified more precisely by the results of our experiment. In the process of creating conical pilot holes using a curette in our experiment, it was assumed that compression effects on the surrounding bone might increase the pullout strength. The experiment confirmed that in both the conical screw and cylindrical screw groups, the mean maximal pullout strength achieved with the 3.2–5 mm curette was significantly greater than that achieved with the same-sized 3.2–5 mm drill bit. However, the mean maximal pullout strength of the 3.2–5 mm curette was lower than that with the 3 mm biopsy needle and the 3.6 mm drill bit, which created smaller pilot holes. Therefore, during pilot hole creation, the drill bit removes bone debris along its flutes, while a conical curette or probe compresses the surrounding bone, resulting in increased pullout strength compared to that achieved using the same size drill bit. However, because the pilot hole volume achieved with the 3.2–5 mm curette was larger than that achieved with the 3–3.6 mm drill bit, meaning more bone was removed, the compressive effect was counteracted.

Our experiment suggests that the volume of the pilot hole is an important factor, with smaller pilot hole volumes leading to increased pullout strength. Therefore, when establishing a pilot hole, it is advisable to avoid creating incorrect paths, as repetitive steps in pilot hole creation can result in oversized pilot hole sizes, leading to decreased pullout strength. Additionally, the diameter of the pilot hole should not exceed the inner diameter of commonly used screws in the surgery, as this difference is also related to decreased pullout strength. Regarding the shape of the screws and pilot holes, the experiment revealed that when using a conical pilot hole, employing conical screws of the same shape resulted in a greater mean maximal pullout strength than using cylindrical screws of different shapes. When extrapolating to real surgical situations, we recommend considering minimally invasive surgery in cases of challenging screw implantation to avoid unnecessarily enlarging the pilot hole size due to repeated creation of incorrect pilot holes. In specific cases where there is a need to improve screw fixation quality, such as in patients with osteoporosis, high-grade spondylolisthesis, unstable spine fractures, etc., minimally invasive surgery could be prioritized. When choosing to perform traditional surgery, using image-guided methods to create smaller diameter pilot holes could be considered to preserve more bone volume around the screw and achieve greater screw fixation strength.

This study has several limitations. First, the research utilized artificial L4 vertebrae as substitutes for real vertebrae. Although this approach eliminates anatomical and bone density differences between individuals, it cannot fully replicate the actual clinical environment. Second, the study only conducted static pullout tests and did not perform other physiologically relevant tests, such as range-of-motion tests or dynamic fatigue tests. Third, this study did not investigate the long-term effects of different pilot hole profiles on screw fixation strength. In clinical settings, both the initial and long-term fixation strength are crucial considerations in clinical surgery following the implantation of pedicle screws. In fact, these two aspects are causally related; without immediate stability in the initial fixation strength after implantation, achieving proper long-term stability becomes challenging. Moreover, in fusion surgeries commonly performed for degenerative spine disorders, the initial fixation strength is of greater concern. This is due to the fact that a stable initial fixation strength can enhance the likelihood of successful bone fusion. Nevertheless, by employing identical procedures and materials throughout the experiment, the study emphasized specificity and reproducibility. Therefore, we believe that the experimental results can still offer spinal surgeons a better understanding of different pilot hole profiles in actual surgeries. Fourth, each group was replicated six times. The relatively small sample size may lead to a lack of statistical significance or an insufficient sample size. However, the six repetitions still enable the calculation of group averages and standard deviations using statistical methods, allowing the authors to assess the precision and reliability of the experiment. Additionally, the study compared various common methods for creating pilot holes in minimally invasive surgery using image guidance and traditional surgery using freehand techniques. However, in practice, there may be slight variations in the sizes of biopsy needles, drill bits, and curettes used by different surgical teams. Some surgeons may also prefer pedicle probes over curettes, introducing differences in size or shape. Nevertheless, our experimental results still effectively illustrate the impact of differences in pilot hole profiles on the pullout strength of screws.

## 5 Conclusion

The strength of this study lies in its comprehensive and systematic comparison of various pilot hole profiles commonly used in minimally invasive and traditional spinal surgery to assess their impact on pullout strength. The diameter of the pilot hole should not exceed the inner diameter of the screw. When using conical pilot holes and screws with the same conical shape, the mean maximal pullout strength surpasses that of cylindrical screws with different shapes. Moreover, pilot holes created for minimally invasive surgery using image-guided techniques exhibit superior pullout strength compared to those utilized in traditional surgery. Therefore, we recommend prioritizing minimally invasive surgery when screw implantation is anticipated to be difficult or when there is a specific need to increase the strength of screw fixation. When opting for traditional surgery, it may be beneficial to use image-guided methods, similar to those employed in minimally invasive procedures, to establish smaller pilot holes and achieve stronger screw fixation.

## Data Availability

The original contributions presented in the study are included in the article/supplementary material, further inquiries can be directed to the corresponding author.
